# Correction: Antimicrobial peptide based magnetic recognition elements and Au@Ag-GO SERS tags with stable internal standards: a three in one biosensor for isolation, discrimination and killing of multiple bacteria in whole blood

**DOI:** 10.1039/c8sc90250j

**Published:** 2018-12-12

**Authors:** Kaisong Yuan, Qingsong Mei, Xinjie Guo, Youwei Xu, Danting Yang, Beatriz Jurado Sánchez, Bingbing Sheng, Chusheng Liu, Ziwei Hu, Guangchao Yu, Hongming Ma, Hao Gao, Christoph Haisch, Reinhard Niessner, Zhengjin Jiang, Haibo Zhou

**Affiliations:** a Institute of Pharmaceutical Analysis , College of Pharmacy , Jinan University , Guangzhou , Guangdong 510632 , China . Email: haibo.zhou@jnu.edu.cn ; Email: jzjjackson@hotmail.com ; Email: tghao@jnu.edu.cn; b School of Medical Engineering , Hefei University of Technology , Tunxi road 193 , Hefei 230009 , China; c Department of Analytical Chemistry , Physical Chemistry and Chemical Engineering , University of Alcala , Alcala de Henares E-28871 , Madrid , Spain; d Shanghai Institute for Advanced Immunochemical Studies , ShanghaiTech University , Shanghai 201210 , China; e Department of Preventative Medicine , Zhejiang Provincial Key Laboratory of Pathological and Physiological Technology , Medical School of Ningbo University , Ningbo , Zhejiang 315211 , China; f The First Affiliated Hospital of Jinan University , Guangzhou , Guangdong 510632 , China; g Institute of Hydrochemistry and Chair for Analytical Chemistry , Technical University of Munich , Marchioninistr. 17, D-81377 , Munich , Germany

## Abstract

Correction for ‘Antimicrobial peptide based magnetic recognition elements and Au@Ag-GO SERS tags with stable internal standards: a three in one biosensor for isolation, discrimination and killing of multiple bacteria in whole blood’ by Kaisong Yuan *et al.*, *Chem. Sci.*, 2018, DOI: 10.1039/c8sc04637a.



## 

The authors regret that the name of one of the authors, Zhengjin Jiang, was displayed incorrectly in the original article. The corrected author list is as shown above.

The Royal Society of Chemistry apologises for these errors and any consequent inconvenience to authors and readers.

